# Supplementary material for: CRELD1 is an evolutionarily-conserved maturational enhancer of ionotropic acetylcholine receptors
Source: eLife. 2018 Nov 7;7:e39649. doi: 10.7554/eLife.39649 (PMC6245729; doi:10.7554/eLife.39649)
Supplement: Transparent reporting form [file elife-39649-transrepform.docx]

***eLife’s* transparent reporting form**

We encourage authors to provide detailed information *within their submission* to facilitate the interpretation and replication of experiments. Authors can upload supporting documentation to indicate the use of appropriate reporting guidelines for health-related research (see [EQUATOR Network](http://www.equator-network.org/%20)), life science research (see the [BioSharing Information Resource](https://biosharing.org/)), or the [ARRIVE guidelines](http://www.plosbiology.org/article/info:doi/10.1371/journal.pbio.1000412) for reporting work involving animal research. Where applicable, authors should refer to any relevant reporting standards documents in this form.

If you have any questions, please consult our Journal Policies and/or contact us: [editorial@elifesciences.org](mailto:editorial@elifesciences.org).

**Sample-size estimation**

- You should state whether an appropriate sample size was computed when the study was being designed
- You should state the statistical method of sample size computation and any required assumptions
- If no explicit power analysis was used, you should describe how you decided what sample (replicate) size (number) to use

No power analysis was used. Sample size was based on standards used in the field. The information of sample size and replication of experiments is provided in figure legends.

Please outline where this information can be found within the submission (e.g., sections or figure legends), or explain why this information doesn’t apply to your submission:

**Replicates**

- You should report how often each experiment was performed
- You should include a definition of biological versus technical replication
- The data obtained should be provided and sufficient information should be provided to indicate the number of independent biological and/or technical replicates
- If you encountered any outliers, you should describe how these were handled
- Criteria for exclusion/inclusion of data should be clearly stated
- High-throughput sequence data should be uploaded before submission, with a private link for reviewers provided (these are available from both GEO and ArrayExpress)

Please outline where this information can be found within the submission (e.g., sections or figure legends), or explain why this information doesn’t apply to your submission:

The number of biological replicates is present in each legend and corresponding figure.

LEVAMISOLE TEST : Figure 1C and 1D, Figure 5B, Figure 6 – figure supplement 1

FLUORESCENCE QUANTIFICATION : Figure 3 +Legend 3G, I.

WESTERN BLOT QUANTIFICATION in *C. elegans* samples: Figure 4A

WESTERN BLOT QUANTIFICATION in differentiated C2C12: Figure 6A-E

RT PCR in differentiated C2C12 : Figure 6G-F, Figure 6 – figure supplement 2

RT PCR in *C. elegans* samples: Figure 4 – figure supplement 1

**Statistical reporting**

- Statistical analysis methods should be described and justified
- Raw data should be presented in figures whenever informative to do so (typically when N per group is less than 10)
- For each experiment, you should identify the statistical tests used, exact values of N, definitions of center, methods of multiple test correction, and dispersion and precision measures (e.g., mean, median, SD, SEM, confidence intervals; and, for the major substantive results, a measure of effect size (e.g., Pearson's r, Cohen's d)
- Report exact p-values wherever possible alongside the summary statistics and 95% confidence intervals. These should be reported for all key questions and not only when the p-value is less than 0.05.

Please outline where this information can be found within the submission (e.g., sections or figure legends), or explain why this information doesn’t apply to your submission:

For all experiments, statistical tests, N values and P values are indicated in the figures and in the figure legends.

LEVAMISOLE TEST : Figure 1C and 1D, Figure 5B, Figure 6 – figure supplement 1ELECTROPHYSIOLOGY DATA: Figure 3B, D, E

FLUORESCENCE QUANTIFICATION : Figure 3 G, I. WESTERN BLOT QUANTIFICATION in *C. elegans* samples: Figure 4A

WESTERN BLOT QUANTIFICATION in differentiated C2C12: Figure 6A-E

RT PCR in differentiated C2C12 : Figure 6G-F, Figure 6 – figure supplement 2

RT PCR in *C. elegans* samples: Figure 4 – figure supplement 1

RT PCR in differentiated C2C12 cells: Figure 6 +Legend 6G-F, Figure S5+Legend S5

RT PCR in *C. elegans* samples: Figure S3 + Legend S3

(For large datasets, or papers with a very large number of statistical tests, you may upload a single table file with tests, Ns, etc., with reference to sections in the manuscript.)

**Group allocation**

- Indicate how samples were allocated into experimental groups (in the case of clinical studies, please specify allocation to treatment method); if randomization was used, please also state if restricted randomization was applied
- Indicate if masking was used during group allocation, data collection and/or data analysis

Please outline where this information can be found within the submission (e.g., sections or figure legends), or explain why this information doesn’t apply to your submission:

This does not apply to our submission

**Additional data files (“source data”)**

- We encourage you to upload relevant additional data files, such as numerical data that are represented as a graph in a figure, or as a summary table
- Where provided, these should be in the most useful format, and they can be uploaded as “Source data” files linked to a main figure or table
- Include model definition files including the full list of parameters used
- Include code used for data analysis (e.g., R, MatLab)
- Avoid stating that data files are “available upon request”

Please indicate the figures or tables for which source data files have been provided:

Source data files have been provided for the following figures:

Figure 1C –source data 1

Figure 1D –source data 2

Figure B,D,E –source data 1

Figure 3G,I –source data 2

Figure 4A –source data 1

Figure 4- figure supplement 1 –source data 2

Figure 5B –source data 1

Figure 6 B,D,E –source data 1

Figure 6 F,G and Figure 6-figure supplement 2–source data 2

Figure 6-figure supplement 1–source data 3
